# Screening of Viral Pathogens from Pediatric Ileal Tissue Samples after Vaccination

**DOI:** 10.1155/2014/720585

**Published:** 2014-03-23

**Authors:** Laura Hewitson, James B. Thissen, Shea N. Gardner, Kevin S. McLoughlin, Margaret K. Glausser, Crystal J. Jaing

**Affiliations:** ^1^The Johnson Center for Child Health and Development, 1700 Rio Grande Street, Austin, TX 78701, USA; ^2^Department of Psychiatry, University of Texas Southwestern, Dallas, TX 75390, USA; ^3^Physical & Life Sciences Directorate, Lawrence Livermore National Laboratory, Livermore, CA 94550, USA; ^4^Computations Directorate, Lawrence Livermore National Laboratory, Livermore, CA 94550, USA

## Abstract

In 2010, researchers reported that the two US-licensed rotavirus vaccines contained DNA or DNA fragments from porcine circovirus (PCV). Although PCV, a common virus among pigs, is not thought to cause illness in humans, these findings raised several safety concerns. In this study, we sought to determine whether viruses, including PCV, could be detected in ileal tissue samples of children vaccinated with one of the two rotavirus vaccines. A broad spectrum, novel DNA detection technology, the Lawrence Livermore Microbial Detection Array (LLMDA), was utilized, and confirmation of viral pathogens using the polymerase chain reaction (PCR) was conducted. The LLMDA technology was recently used to identify PCV from one rotavirus vaccine. Ileal tissue samples were analyzed from 21 subjects, aged 15–62 months. PCV was not detected in any ileal tissue samples by the LLMDA or PCR. LLMDA identified a human rotavirus A from one of the vaccinated subjects, which is likely due to a recent infection from a wild type rotavirus. LLMDA also identified human parechovirus, a common gastroenteritis viral infection, from two subjects. Additionally, LLMDA detected common gastrointestinal bacterial organisms from the *Enterobacteriaceae*, *Bacteroidaceae*, and *Streptococcaceae* families from several subjects. This study provides a survey of viral and bacterial pathogens from pediatric ileal samples, and may shed light on future studies to identify pathogen associations with pediatric vaccinations.

## 1. Introduction

Rotavirus is the most common cause of severe diarrhea among infants and young children [1]. Prior to the introduction of rotavirus vaccines, rotavirus infection was estimated to cause approximately 2.7 million cases of severe gastroenteritis in children, almost 60,000 hospitalizations, and around 37 deaths each year in the USA alone [2]. Three vaccines against rotavirus have been developed: Rotashield (Wyeth-Lederle Vaccines and Pediatrics, [3]), RotaTeq (Merck, [4]), and Rotarix (GlaxoSmithKline, [5]). Rotashield, a rhesus-based tetravalent rotavirus vaccine, was recommended for routine vaccination of US infants in 1999 [6] but was withdrawn from the US market within 1 year of its introduction because of its association with intussusception [7]. RotaTeq, a human-bovine reassortant rotavirus vaccine [8], was recommended for vaccination of US infants in 2006 [9] with 3 doses administered orally at ages 2, 4, and 6 months [10]. In 2008, Rotarix, a monovalent vaccine based on an attenuated human rotavirus [11], was licensed in the USA for pediatric use as a 2-dose series and recommended for vaccination of US infants in June 2008 [12]. Since the introduction of rotavirus vaccines, there has been a dramatic reduction in the incidence and severity of rotavirus infections both in the US and globally [13–16].

During the course of developing novel virus detection techniques, researchers at the San Francisco Blood Research Systems Institute and Lawrence Livermore National Laboratory (LLNL) unexpectedly identified nucleic acids from an adventitious virus in Rotarix [17]. The detected virus shared 98% homology with porcine circovirus-1 (PCV-1) and covered the complete circular genome [17]. PCV infection is common in pigs and the virus is often detected in human stool samples [18] but is not believed to cause illness among humans [19–21]. Contamination of Rotarix with PCV-1 was subsequently confirmed by the vaccine manufacturer. In March 2010, in light of these findings, the US Food and Drug Administration (FDA) recommended temporarily suspending the use of Rotarix [22]. On May 6, 2010, the FDA reported preliminary findings that the RotaTeq vaccine also contained detectable PCV material [23].

On May 7, 2010, the FDA Vaccines and Related Biological Products Advisory Committee (VRBPAC) met to review whether the contaminated rotavirus vaccines could pose risks to human health. The committee concluded that based on the available evidence, the hypothetical risk of PCV infection among humans does not outweigh the observed benefits of rotavirus vaccines in preventing severe acute gastroenteritis among infants. The committee expressed reassurance that the detection of DNA and DNA fragments from PCV in rotavirus vaccines was not likely to cause harm to humans and recommended that information on this topic be provided to parents prior to vaccination. The committee did, however, recommend that the vaccine manufacturers work to develop rotavirus vaccines free of PCV1 and PCV2 contaminants. On May 14, 2010, the FDA issued a recommendation for pediatricians to resume use of Rotarix and to continue use of RotaTeq [24]. Subsequent testing by the vaccine manufacturers identified that the PCV material was introduced into both rotavirus vaccines through porcine-derived trypsin, a reagent used in the cell-culture growth process of vaccine production, commencing very early in the development process [17, 25]. The use of cells or biological products from other species in the production of vaccines can lead to leakage of cellular DNA and the introduction of noninfectious proviral DNA [17].

While the recent publicity about potential safety concerns over rotavirus vaccines does not appear to have had a negative impact on vaccine administration practices of most physicians, it has raised safety concerns among some parents [26]. The goal of this study was to use a novel DNA detection technology, the Lawrence Livermore Microbial Detection Array (LLMDA), [27, 28] to determine whether viral or bacterial DNA or DNA fragments, including PCV, could be detected in ileal tissue samples from children following vaccination with rotavirus vaccines.

The LLMDA is a nucleic acid detection technology that contains a total of 388,000 probes, designed to detect 2,200 viral species (38,000 target sequences) and 900 bacterial species (3,500 target sequences). This microarray uses long (50–65 base-pair) oligonucleotide probes to enable sensitive detection of known viral and bacterial species and the detection of divergent species with homology to sequenced organisms. The array data is analyzed using a novel composite likelihood maximization method to predict the strains and species that are most likely present in a sample. Each target detected has a log likelihood score, which is estimated from the BLAST similarity scores of the probes to each of the possible target sequences, together with the probe sequence complexity and other covariates derived from the BLAST results. Targets are color-coded and grouped by taxonomic family. This array has been used to detect a wide array of viral infections from various clinical samples [27].

Though various nucleic acid detection technologies including TaqMan PCR and Luminex bead based systems are able to identify selected pathogens at the species or strain level rapidly, they do not have the capability to provide broad spectrum detection about known or novel organisms. While sequencing provides the most in-depth information to characterize a microbial genome, the costs, labor, and time associated with library preparation, sequencing, bioinformatic analysis, and data storage may be prohibitive when analyzing many isolates to screen for pathogens. Microarrays provide a means for broad surveillance of sequenced pathogens with assay time and cost close to PCR assays. In this study, we first used the LLMDA to screen for viral and bacterial pathogens in human ileal samples, and then used PCR to confirm the microarray findings.

## 2. Materials and Methods

### 2.1. Ethics Statement

This study was approved by the Austin Multi-Institutional Review Board (AMIRB). Subjects were scheduled to undergo a diagnostic ileocolonoscopy for chronic GI symptoms during the period from January 2008 to December 2010. They were recruited from a single pediatric gastroenterology clinic and written informed consent was received from the parent or guardian of all subjects prior to enrollment. Twenty-one subjects aged 16 to 52 months were included in this study and represented 15 males and 6 females. Subjects were (i) vaccinated against rotavirus (*n* = 9) using one of two rotavirus vaccines (Rotarix or RotaTeq); (ii) vaccinated but not against rotavirus (*n* = 8); or (iii) unvaccinated (*n* = 4). Subject demographics and vaccine status are shown in Table 1.

### 2.2. Sample Collection and Processing

A pinch biopsy from the terminal ileum was collected using a standard disposable forceps biopsy device, in accordance with routine diagnostic biopsy protocol. Each biopsy retrieved was immediately dissected so that at least half of the biopsy was fixed for subsequent histological examination for clinical pathology. The remaining sample was placed directly into RNAlater (Qiagen Inc., Valencia, CA) for between 24 and 48 hours and subsequently stored at −80°C until processing. All samples were coded and were blinded in regard to vaccination status. Samples were shipped to LLNL on dry ice. One sample of the reportedly PCV-contaminated RotaTeq live, oral, pentavalent vaccine (lot 0147Z) was also included in the analyses. The PCV-contaminated Rotarix was not available for analysis.

### 2.3. Nucleic Acid Extraction from Human Ileum and Vaccine Samples

#### 2.3.1. Extraction from Human Ileum

One ileum sample was extracted per patient. Each ileum was roughly 20 mg and cut into approximately four smaller pieces prior to being placed in a 2 mL bead beating tube containing 0.5 g of 1.0 mm zirconia beads and 500 *μ*L of chilled Hank's buffered salt solution. The tubes were bead beat for 30 sec at 25 speed. Following bead beating the samples were clarified by centrifuging for 5 min at 15,000 ×g. The supernatant was transferred to a new 1.5 mL tube to continue nucleic acid extraction. Due to the small amount of ileal tissue available for this study, no DNase treatment or filtration to remove bacterial or host cells was performed. Nucleic acids were extracted using the Qiagen QIAamp UltraSens Virus Kit (Qiagen) according to the manufacturer's instructions. Following extraction the nucleic acid concentration was determined using the Invitrogen Qubit fluorometer (Invitrogen, Grand Island, NY). Approximately 400 ng of DNA and 1.4 *μ*g of RNA were obtained from each ileum sample after extraction.

#### 2.3.2. Extraction from RotaTeq Vaccine

A RotaTeq vaccine sample was extracted for analysis. One dose contained 2 mL of the vaccine; therefore, two 1 mL extractions were performed. Each 1 mL extraction was performed using the QIAamp UltraSens Virus Kit following the manufacturer's protocol. Following extraction each vaccine sample was combined and nucleic acid concentration was determined using a Qubit fluorometer.

### 2.4. Microarray Processing

#### 2.4.1. Whole Genome Amplification and Purification

The extracted ileum and vaccine samples were whole genome amplified using a random amplification protocol as described previously [26]. Briefly, 50 ng of DNA from each terminal ileal sample and 10 ng of DNA from the RotaTeq vaccine sample were used in the amplification procedure. The amplification procedure was performed by incubating each sample with 1 *μ*L of random primer 5′-GATGAGGGAAGATGGGGNNNNNNNNN-3′ (100 pmole/*μ*L) for 2 min at 85°C and immediately placed on ice for 2 min. To each reaction, 4 *μ*L 5x Superscript III buffer, 1 *μ*L dNTP (12.5 mM), 2 *μ*L DTT (0.1 M), 1 *μ*L Invitrogen Superscript III reverse transcriptase, and 1 *μ*L Ultrapure DEPC water (Invitrogen) were added. The samples were placed in a Tetrad PTC-225 thermal cycler (MJ Research, Quebec, Canada) with the following conditions: 25°C for 10 min, 42°C for 2 hours, and 70°C for 5 min. Following first strand synthesis, each 20 *μ*L sample was mixed with 2.4 *μ*L 10x Klenow buffer (New England Biolabs, Ipswich, MA) and 0.5 *μ*L 12.5 mM dNTP (New England Biolabs). Next, the samples were incubated for 2 min at 85°C and immediately placed on ice for 2 min. Lastly, 1 *μ*L Klenow buffer was added to the samples and allowed to incubate at 37°C for 60 min followed by 70°C for 20 min.

Samples were amplified by combining 5 *μ*L of the double-stranded cDNA product with 5 *μ*L 10x Sigma Taq buffer, 1 *μ*L dNTP (12.5 mM), 1 *μ*L primer 5′-GATGAGGGAAGATGGGG-3′ (100 pmole/*μ*L), 1 *μ*L Sigma KlenTaq LA polymerase, and 37 *μ*L water. Reactions were placed in a thermocycler (Tetrad Thermal Cycler, MJ Research, Quebec, Canada) with the following conditions: 94°C for 2 min; 40 cycles of 94°C for 30 sec, 50°C for 1 min, and 68°C for 1 min; and 72°C for 10 min. Amplified samples were purified using the Qiaquick PCR Purification Columns (Qiagen) according to manufacturer's instructions. Samples were eluted in 40 *μ*L of Buffer EB from the Qiagen kit and nucleic acid concentration determined by Qubit fluorometer.

#### 2.4.2. Microarray Hybridization

We used the LLMDA v2 for analysis of viral or bacterial content from the tissue or vaccine samples. This array contains 388,000 probes to detect all sequenced viruses and bacteria that we sequenced before April of 2007 [28]. Additionally, we analyzed a subset of the samples using a multiplexed format of the LLMDA v2 printed on the Roche NimbleGen (Roche NimbleGen, Madison, WI) 4 × 72 K array format. Samples 1–8 and 10 were run on the 4 × 72 K format of the LLMDA. The other samples and RotaTeq were run on the 388 K format of the LLMDA.

For each sample, 1 *μ*g of amplified product was fluorescently labeled using the One-Color DNA Labeling Kit (Roche NimbleGen) according to the recommended protocols. The DNA was purified after labeling and hybridized using the NimbleGen Hybridization Kit to the LLMDA according to manufacturers' instructions. The microarrays were allowed to hybridize for 17 hours and washed using the NimbleGen Wash Buffer Kit according to manufacturer's instructions. Microarrays were scanned on an Axon GenePix 4000B 5 *μ*m scanner (Molecular Devices, Sunnyvale, CA). The scanned tiff image files were aligned using the NimbleScan Version 2.4 software and pair text files were exported for data analysis.

### 2.5. Microarray Data Analysis

Data was analyzed using the automated LLMDA analysis algorithm—composite likelihood maximization algorithm [28]. A threshold of 99% was used in the data analysis to analyze only the probes with signal intensity above 99% of random controls. Random controls are probes that do not hybridize to any known target sequences and were designed to match the overall GC content and thermodynamics of the target probes.

### 2.6. PCR Primer Design for Confirmation of Viral Pathogens from the LLMDA

Taqman signatures were designed using the run Primux triplet script that is part of the PriMux software [29] for the viruses detected in ileum samples 5, 7, 9, 11, and 14. Target sets were comprised of the available complete sequences for the following viruses: Torque teno virus (TTV)-like minivirus (6 genomes, validation for sample 7), human parechoviruses (44 genomes, validation for sample 9), small anelloviruses and Torque teno midi viruses (TTMV) (20 genomes, samples 11 and 14), echovirus 9 (7 genomes, validation for sample 5), and rotavirus A (7077 sequences, all segments, for sample 5). Predicted targets were identified using simulate_PCR.pl (submitted, https://sourceforge.net/projects/simulatepcr) based on comparison to the LLNL large (48 GB) internal database of all available finished and assembled microbial genomes from NCBI, multiple public and university sequencing centers (e.g., Broad, JCVI, IMG, Sanger, Singapore, etc.), and from collaborators, currently over 48 GB of sequence data. From the multiple signatures designed for each target set, one was selected that was predicted to detect the virus and its near neighbors that were reported by LLMDA results for that sample. Primer sequences and expected amplicon sizes can be seen in Table 2. For PCR to detect PCV-2, an 84 bp PCR assay from previous studies of the RotaTeq vaccine [25] was used.

### 2.7. PCR Analysis

PCR primers were ordered through Integrated DNA Technologies. Each real-time PCR reaction consisted of 2.5 *μ*L 10x PCR buffer (Invitrogen), 1 *μ*L forward/reverse primer mix (10 *μ*M), 1.75 *μ*L MgCl_2_ (50 mM), 1 *μ*L BSA (2 *μ*g/*μ*L), 0.5 *μ*L dNTPs (10 mM), 0.25 *μ*L Invitrogen Platinum Taq polymerase (5 U/*μ*L), and 13 *μ*L water. All reactions were carried out on a Tetrad PTC-225 thermal cycler with the following conditions: 95°C for 3 min; 40 cycles of 95°C for 20 sec, 60°C for 30 sec, and 72°C for 30 sec; and 72°C for 2 min. Reactions with the RotaTeq vaccine and other controls were run with 1 ng of total DNA or cDNA, while reactions with ileum samples were run with 10 ng of total DNA or cDNA. Each sample was run in duplicate. The PCR products from the duplicate reactions were then combined and run on a 4% agarose gel.

## 3. Results

### 3.1. Microbial Detection Array Results

**Figure 1:**
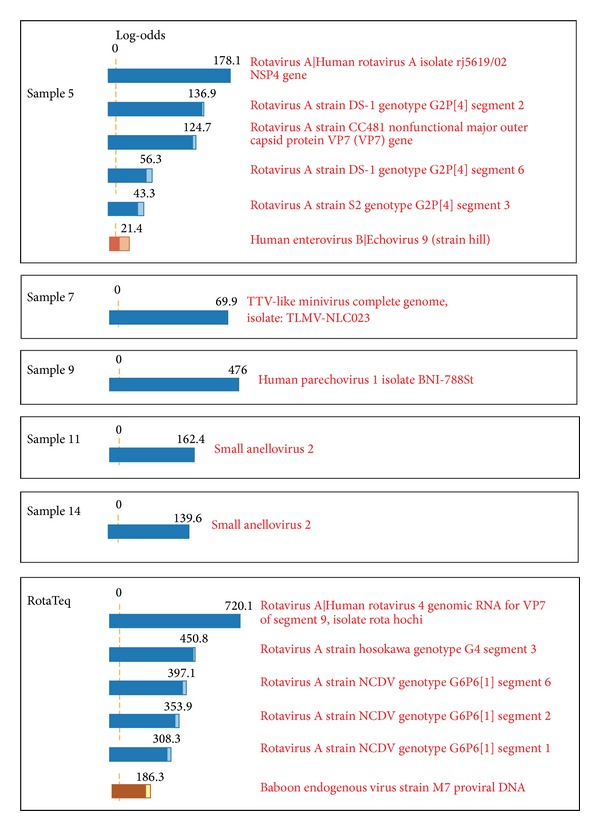
LLMDA viral results from human ileum samples and RotaTeq. Microarray data were analyzed using the composite likelihood maximization method developed at Lawrence Livermore National Laboratory [28]. The log likelihood for each of the possible targets is estimated from the BLAST similarity scores of the probe and target sequences, together with the probe sequence complexity and other covariates derived from the BLAST results [28].

The viruses detected from the samples by the LLMDA array are shown in Figure 1. Microarray data analysis parameters were set to give both bacterial and viral results with probe signal intensity above 99% of random control probes. In sample 5, LLMDA detected probes that hybridized to several segments of the human rotavirus A and an echovirus 9. The detected rotavirus segments all appear to be from human origin. LLMDA also identified a human parechovirus 1 from sample 9. Small anellovirus 2 was detected in samples 11 and 14. TTV-like minivirus was detected in sample 7. Human endogenous retroviruses (HERVs) were detected in most ileal samples (data not shown). This is likely due to the residual human genomic DNA present in the samples. No other viral targets were identified from other ileal samples. A summary of the viral results is shown in Table 3. LLMDA identified several segments of human rotavirus A (segments 7, 9, and 3) from the RotaTeq vaccine (Figure 1). Several bovine rotavirus sequence segments including segment 1, 2, and 6 were also detected. Additionally, a baboon endogenous virus strain M7 was detected, likely due to the monkey cell line in which RotaTeq was produced from.

Additionally, LLMDA also detected bacteria from some of the ileum samples, summarized in Table 4.* Bacteroides* species were identified in samples 1, 5, 6, 15, 18, and 20. Plasmids from the* Shigella* species were detected in samples 5 and 15.* Streptococcus agalactiae* was detected in sample 15, and* Streptomyces coelicolor* was detected in sample 20.

### 3.2. PCR Assay to Confirm Microarray Results

**Figure 2:**
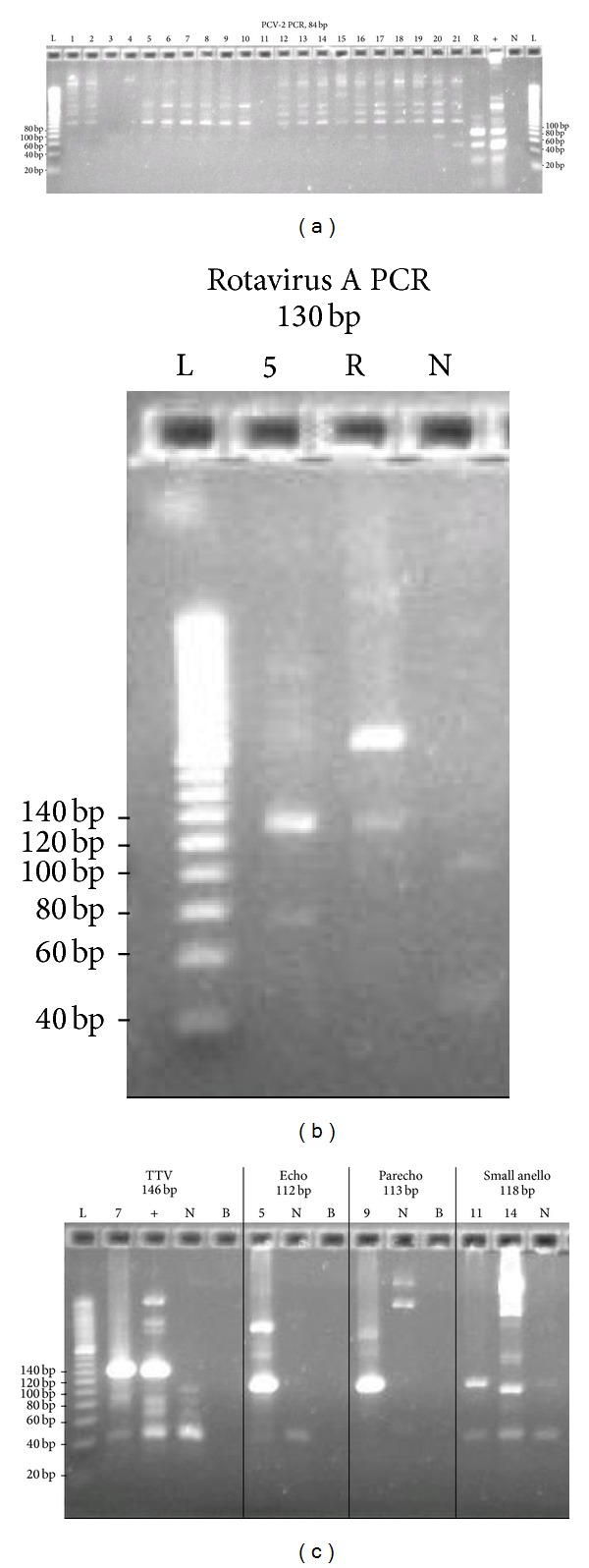
Results of PCR confirmation of PCV-2, rotavirus A, parechovirus, echovirus, TTV, and small anellovirus from selected samples. (a) PCV-2 PCR. Lanes 1–21 are human ileum samples. R: RotaTeq vaccine; +: PK-15 cell control; N: NTC; L: 20 bp ladder. Both RotaTeq and positive control gave expected size at 84 bp. (b) Rotavirus A PCR. Sample 5 and RotaTeq gave expected band at 130 bp. No product was detected in NTC. (c) PCR results of TTV, Echovirus, parechovirus, and small anellovirus. B: blank. Sample 7 and a positive control from a previous study gave expected band size at 146 bp. Echovirus PCR from sample 5 detected an expected band size at 112 bp. Parechovirus PCR from sample 9 detected an expected band size at 113 bp. Small anellovirus PCR from samples 11 and 14 produced expected band size at 118 bp.

The viruses detected by microarrays and the negative PCV results from microarrays were all confirmed by PCR assays (Figure 2). The PCV-2 PCR results are shown in Figure 2(a). None of the human ileum samples showed any band at 84 bp, the expected size of the PCR amplicon. Both the RotaTeq sample and a positive control sample from an ATCC cell line (PK-15) showed bands around 84 bp on the gel.

PCR of rotavirus A from sample 5 and the RotaTeq vaccine gave the expected band at 140 bp (Figure 2(b)). PCR of TTV-like minivirus from sample 7 and a positive control from a previous study detected the expected band at 112 bp. Additionally, echovirus 9 PCR from sample 5 detected an expected product at 112 bp. PCR of human parechoviruses 1 from sample 9 detected the expected product at 113 bp. Small anelloviruses PCR from samples 11 and 14 produced expected band size at 118 bp (Figure 2(c)).

## 4. Discussion

We analyzed 21 human terminal ileum samples, obtained from children undergoing routine colonoscopy, for the presence of any viral and bacterial pathogens, and evaluated any association of specific pathogens with vaccination. The samples were analyzed on the LLMDA v2, which contains DNA probes to detect all sequenced viruses and bacteria [28]. The viruses detected by the LLMDA were subsequently confirmed by PCR assays. The intestinal mucosa is an ideal tissue for the study of virus—host interactions, as it is the site of ileal Peyer's patches composed of lymphoid cells, which are important in immune surveillance of the intestinal lumen. Ileal samples were collected from children that had been (i) fully vaccinated including against rotavirus; (ii) previously vaccinated but not against rotavirus; or (iii) completely unvaccinated.

Overall, no correlation between specific pathogens and vaccination status was identified from this study, nor was a correlation identified between pathogens and vaccination of rotavirus vaccines. PCV was not detected in any ileal samples either by microarray or PCR analyses.

A sample of the RotaTeq vaccine that had been previously shown to contain PCV DNA was included for analysis but the PCV-contaminated Rotarix vaccine was not available for analysis. The sample of RotaTeq vaccine tested positive for rotavirus A and baboon endogenous virus, as previously reported by Victoria and colleagues [17]. The origin of the baboon endogenous virus is assumed to be related to the African green monkey-derived Vero cell line used in its manufacture and cross-hybridization of its endogenous retroviruses to the baboon endogenous retrovirus probes [17].

Microarray analysis did not detect PCV from the RotaTeq vaccine, which confirmed the previous results from Victoria et al. that LLMDA detected PCV from Rotarix but did not detect PCV from the RotaTeq vaccine [17]. However, PCV2 in RotaTeq vaccine was detected by PCR assays. RotaTeq contained small PCV-1 and PCV-2 genome fragments but did not contain detectable larger portions of PCV genomes [30]. Studies have shown that the amount of PCV in RotaTeq was about 4000 times lower than the PCV in Rotarix, with the PCV in RotaTeq being barely detectable [25, 31, 32]. A case study by Ranucci et al. has reported that the concentration of PCV-2 DNA fragment in clinical consistency lots was in the range of below limit of detection to 6.4 × 10^3^ copies/mL when measured by QPCR, and that PCV1 was below limit of detection (0.1–0.8 × 10^3^ copies/mL) [30]. The current study showed that the PCV-2 signal was close to or above the limit of detection of PCR, but below detection limit of LLMDA. PCV was not detected in any ileum samples either by microarray or PCR analyses. It is also likely that the PCV fragments from the RotaTeq vaccine have already been eliminated from the body, thus no PCV remains in the ileal samples.

Human rotavirus A was detected in one ileum sample (sample 5) by microarray and confirmed by PCR assay. This sample came from a fully vaccinated child and the infection likely from a recent rotavirus infection. It is unlikely that this is the remaining rotavirus since the child was vaccinated with RotaTeq about two years ago. RotaTeq (Merck) is a pentavalent vaccine that contains five live-attenuated strains with genotypes G1P[5], G2P[5], G3P[5], G4P[5], and G6P[8], derived through laboratory reassortment of human rotavirus strains with a bovine G6P[5] rotavirus strain (WC3) [33]. The LLMDA detected several segments of the virus, all from human origin. The genotype detected in this sample, G2P[4], is not a vaccine genotype and it has been previously identified in G1P[8] vaccinated patients [34].

In the same sample 5, echovirus 9 was also identified. Additionally, a closely related human parechovirus 1 was identified in sample 9. Both the echovirus 9 and human parechovirus 1 detection by microarray were confirmed by echovirus 9 and human parechovirus 1 specific PCR assays. Echovirus is a subspecies of the human enterovirus B found in the gastrointestinal tract. Human enteroviruses cause mild, gastrointestinal, or respiratory illness [35] and are commonly spread such that more than 95% of children are infected within two to five years of age [35, 36]. Nyström et al. have recently found human enterovirus species B in ileocecal Crohns' disease [37], suggesting that this viral species could play a role in Crohn's disease onset or progression.

Small anellovirus 2 was detected in two patient samples. Small anellovirus 2 is also referred to as Torque teno midi virus (TTMV). TTMV and TTVs are ubiquitous in >90% of adults worldwide but no human pathogenicity of TTV has been fully established [38, 39]. No significant viruses were identified in any other samples. Analysis of a larger number of ileum samples will help further identify any additional coinfecting pathogens, as well as the frequency of occurrence of these pathogens.

The application of the LLMDA technology provides an effective means to survey vaccines for the presence of adventitious agents. In this study, LLMDA did not detect any PCV DNA sequences from the pediatric ileal samples; however, LLMDA detected wild type rotavirus, human enterovirus B, small anellovirus, TTMV, and common gastrointestinal bacteria including* Bacteroides*,* Shigella,* and* Streptococcus* from some samples, suggesting that LLMDA could be used as a tool to monitor the effectiveness of rotavirus vaccines and to detect reinfections and coinfections with other gastrointestinal viruses or bacteria that could cause pediatric gastrointestinal problems.

## 5. Conclusions

We analyzed 21 children ileal samples from colonoscopy on the LLMDA array to screen for bacterial and viral pathogens and possible adventitious agents that could be associated with vaccination. We detected a wild type rotavirus, parechovirus, and several common gastrointestinal bacterial agents,* Bacteroides*,* Shigella,* and* Streptococcus* from several ileal samples. This study shows that the broad spectrum technology, such as the LLMDA, could be used as a surveillance tool for vaccine safety and effectiveness.

## Figures and Tables

**Table 1:** Subject demographics. Demographics of study subjects include gender, year of birth, vaccination status, and age in months at sample collection. Rota^−^, vaccinated but not against rotavirus; Rota^+^, fully vaccinated including against rotavirus.

Subject ID	Sex	Year of birth	Vaccination status	Age (months) at collection
1	Male	2007	Vaccinated/Rota^−^	16.3
2	Female	2006	Unvaccinated	21.8
3	Female	2007	Vaccinated/Rota^+^	17.3
4	Female	2006	Vaccinated/Rota^−^	23.9
5	Male	2006	Vaccinated/Rota^+^	27.9
6	Female	2006	Vaccinated/Rota^−^	33.2
7	Male	2006	Unvaccinated	31.7
8	Female	2006	Vaccinated/Rota^+^	29.4
9	Female	2006	Vaccinated/Rota^−^	30
10	Male	2006	Vaccinated/Rota^+^	34.2
11	Male	2006	Vaccinated/Rota^−^	41.3
12	Male	2006	Unvaccinated	47.5
13	Male	2007	Vaccinated/Rota^−^	33
14	Male	2006	Vaccinated/Rota^−^	41.1
15	Male	2007	Vaccinated/Rota^+^	36.1
16	Male	2006	Unvaccinated	47.3
17	Male	2006	Vaccinated/Rota^+^	52.3
18	Male	2006	Vaccinated/Rota^−^	46.5
19	Male	2006	Vaccinated/Rota^+^	50.9
20	Male	2008	Vaccinated/Rota^+^	32.2
21	Male	2006	Vaccinated/Rota^+^	47.8

**Table 2:** PCR assay primer sequences and expected amplicon sizes. The primers for echovirus, TTV like minivirus, small anellovirus, parechovirus, and rotavirus A were designed by LLNL as part of this study. The primers for PCV-2 were obtained from McClenahan et al. [25].

Oligo name	Sequence	Amplicon Size
Echovirus9_F	GCC CCT GAA TGC GGC TAA	112 bp
Echovirus9_R	AAA CAC GGA CAC CCA AAG TAG T	
TTV-like mini_F	CGA ATG GCT GAG TTT ATG CC	146 bp
TTV-like mini_R	GTT TCT TGC CCG TTC CGC	
Small anello_F	CTG AGT TTA CCC CGC TAG AC	118 bp
Small anello_R	CCG AAT TGC CCC TAG ACC	
Parechovirus_F	CCC AYG AAG GAT GCC CAG	113 bp
Parechovirus_R	TTG GCC CAC TAG ACG TTT T	
Rotavirus_seg10_F	CCA ADW GAA GTG ACY GCA	130 bp
Rotavirus_seg10_R	GCG ATA TGR YTG ACT DTG GCT	
PCV2_F	AGCAATCAGAYCCCGTTG	84 bp
PCV2_R	CCAAGGAVGTAATCCTCCGATA	

**Table 3:** Summary of vaccination status, viruses detected by microarray, and PCR confirmation results from human ileal samples. (+, vaccinated; −, unvaccinated).

Sample ID	Vaccination status	LLMDA viral results	PCR confirmation
1	+	Not detected	
2	−	Not detected	
3	+	Not detected	
4	+	Not detected	
5	+	Human rotavirus A human echovirus 9	Yes
6	+	Not detected	
7	−	TTV-like minivirus	Yes
8	+	Not detected	
9	+	Human parechovirus	Yes
10	+	Not detected	
11	+	Small anellovirus 2	Yes
12	−	Not detected	
13	+	Not detected	
14	+	Small anellovirus 2	Yes
15	+	Not detected	
16	−	Not detected	
17	+	Not detected	
18	+	Not detected	
19	+	Not detected	
20	+	Not detected	
21	+	Not detected	

**Table 4:** Bacterial sequences detected from the human ileum samples by the LLMDA array. Microarray data was analyzed using the CliMax software as described [28]. (+, vaccinated; −, unvaccinated).

Sample ID	Vaccination status	Bacterial results
1	**+**	*Bacteroides thetaiotaomicron *
		*Bacteroides coprocola *

2	−	Not detected

3	**+**	Not detected

4	**+**	Not detected

5	**+**	*Shigella sonnei *
		*Klebsiella pneumoniae *
		*Shigella dysenteriae *
		*Bacteroides intestinalis *

6	**+**	*Bacteroides fragilis *
		*Bacteroides vulgatus *
		*Bacteroides plebeius *

7	−	Not detected

8	**+**	Not detected

9	**+**	Not detected

10	**+**	Not detected

11	**+**	Not detected

12	−	Not detected

13	**+**	Not detected

14	**+**	Not detected

15	**+**	*Shigella sonnei *
		*Bacteroides thetaiotaomicron *

16	−	*Streptococcus agalactiae *

17	**+**	Not detected

18	**+**	*Bacteroides thetaiotaomicron *

19	**+**	Not detected

20	**+**	*Bacteroides thetaiotaomicron *
		*Streptomyces coelicolor *

21	**+**	Not detected

## References

[1] Dennehy PH (2000). Transmission of rotavirus and other enteric pathogens in the home. *The Pediatric Infectious Disease Journal*.

[2] Fischer TK, Viboud C, Parashar U (2007). Hospitalizations and deaths from diarrhea and rotavirus among children <5 years of age in the United States, 1993–2003. *The Journal of Infectious Diseases*.

[3] Kapikian AZ, Hoshino Y, Chanock RM, Perez-Schael I (1996). Efficacy of a quadrivalent rhesus rotavirus-based human rotavirus vaccine aimed at preventing severe rotavirus diarrhea in infants and young children. *The Journal of Infectious Diseases*.

[4] Matson DO (2006). The pentavalent rotavirus vaccine, RotaTeq. *Seminars in Pediatric Infectious Diseases*.

[5] Bernstein DI, Ward RL (2006). Rotarix: development of a live attenuated monovalent human rotavirus vaccine. *Pediatric Annals*.

[6] CDC (1999). Rotavirus vaccine for the prevention of rotavirus gastroenteritis among children. *Morbidity and Mortality Weekly Report*.

[7] CDC (1999). Withdrawal of rotavirus vaccine recommendation. *Morbidity and Mortality Weekly Report*.

[8] Heaton PM, Goveia MG, Miller JM, Offit P, Clark HF (2005). Development of a pentavalent rotavirus vaccine against prevalent serotypes of rotavirus gastroenteritis. *The Journal of Infectious Diseases*.

[9] CDC (2006). Prevention of rotavirus gastroenteritis among infants and children. Recommendations of the Advisory Committee on Immunization Practices (ACIP). *Morbidity and Mortality Weekly Report*.

[10] CDC (2007). Recommended immunization schedules for persons aged 0–18 years—United States. *Morbidity and Mortality Weekly Report*.

[11] de Vos B, Vesikari T, Linhares AC (2004). A rotavirus vaccine for prophylaxis of infants against rotavirus gastroenteritis. *The Pediatric Infectious Disease Journal*.

[12] CDC (2009). Prevention of rotavirus gastroenteritis among infants and children. Recommendations of the Advisory Committee on Immunization Practices (ACIP). *Morbidity and Mortality Weekly Report*.

[13] Curns AT, Steiner CA, Barrett M, Hunter K, Wilson E, Parashar UD (2010). Reduction in acute gastroenteritis hospitalizations among US children after introduction of rotavirus vaccine: analysis of hospital discharge data from 18 US States. *The Journal of Infectious Diseases*.

[14] O’Ryan M, Diaz J, Mamani N, Navarrete M, Vallebuono C (2007). Impact of rotavirus infections on outpatient clinic visits in Chile. *The Pediatric Infectious Disease Journal*.

[15] Tate JE, Patel MM, Steele AD (2010). Global impact of rotavirus vaccines. *Expert Review of Vaccines*.

[16] Patel MM, Steele D, Gentsch JR, Wecker J, Glass RI, Parashar UD (2011). Real-world impact of rotavirus vaccination. *The Pediatric Infectious Disease Journal*.

[17] Victoria JG, Wang C, Jones MS (2010). Viral nucleic acids in live-attenuated vaccines: detection of minority variants and an adventitious virus. *Journal of Virology*.

[18] Li L, Kapoor A, Slikas B (2010). Multiple diverse circoviruses infect farm animals and are commonly found in human and chimpanzee feces. *Journal of Virology*.

[19] Ellis JA, Wiseman BM, Allan G (2000). Analysis of seroconversion to *Porcine circovirus* 2 among veterinarians from the United States and Canada. *Journal of the American Veterinary Medical Association*.

[20] Allan GM, Mcneilly F, Mcnair I (2000). Absence of evidence for *Porcine circovirus* type 2 in cattle and humans, and lack of seroconversion or lesions in experimentally infected sheep. *Archives of Virology*.

[21] Hattermann K, Roedner C, Schmitt C, Finsterbusch T, Steinfeldt T, Mankertz A (2004). Infection studies on human cell lines with *Porcine circovirus* type 1 and *Porcine circovirus* type 2. *Xenotransplantation*.

[22] FDA http://www.fda.gov/NewsEvents/Newsroom/PressAnnouncements/ucm205625.htm.

[23] FDA Vaccines and Related Biological Products Advisory Committee Meeting Background Material. http://www.fda.gov/BiologicsBloodVaccines/Vaccines/ApprovedProducts/ucm211101.htm.

[24] FDA Update on Recommendations for the Use of Rotavirus Vaccines. http://www.fda.gov/BiologicsBloodVaccines/Vaccines/ApprovedProducts/ucm212140.htm.

[25] McClenahan SD, Krause PR, Uhlenhaut C (2011). Molecular and infectivity studies of *Porcine circovirus* in vaccines. *Vaccine*.

[26] Payne DC, Humiston S, Opel D (2011). A multi-center, qualitative assessment of pediatrician and maternal perspectives on rotavirus vaccines and the detection of *Porcine circovirus*. *BMC Pediatrics*.

[27] Erlandsson L, Rosenstierne MW, McLoughlin K, Jaing C, Fomsgaard A (2011). The microbial detection array combined with random Phi29-amplification used as a diagnostic tool for virus detection in clinical samples. *PLoS ONE*.

[28] Gardner SN, Jaing CJ, McLoughlin KS, Slezak TR (2010). A microbial detection array (MDA) for viral and bacterial detection. *BMC Genomics*.

[29] Hysom D,  Naraghi-Arani P, Elsheikh M, Carrillo AC, Williams PL, Gardner SN (2012). Skip the alignment: degenerate, multiplex primer and probe design using k-mer matching instead of alignments. *PLoS ONE*.

[30] Ranucci CS, Tagmyer T, Duncan P (2011). Adventitious agent risk assessment case study: evaluation of RotaTeq for the presence of *Porcine circovirus*. *PDA Journal of Pharmaceutical Science and Technology*.

[31] Gilliland SM, Forrest L, Carre H (2012). Investigation of *Porcine circovirus* contamination in human vaccines. *Biologicals*.

[32] Baylis SA, Finsterbusch T, Bannert N, Blumel J, Mankertz A (2011). Analysis of *Porcine circovirus* type 1 detected in Rotarix vaccine. *Vaccine*.

[33] Payne DC, Wikswo M, Parashar UD, Roush SW, McIntyre L, Baldy LM (2012). Manual for the surveillance of vaccine-preventable diseases. *Rotavirus*.

[34] Antunes H, Afonso A, Iturriza M (2009). G2P[4] the most prevalent rotavirus genotype in 2007 winter season in an European non-vaccinated population. *Journal of Clinical Virology*.

[35] Stanway G, Joki-Korpela P, Hyypia T (2000). Human parechoviruses—biology and clinical significance. *Reviews in Medical Virology*.

[36] Joki-Korpela P, Hyypia T (2001). Parechoviruses, a novel group of human picornaviruses. *Annals of Medicine*.

[37] Nyström N, Berg T, Lundin E (2013). Human enterovirus species B in ileocecal Crohn's disease. *Clinical and Translational Gastroenterology*.

[38] Hino S, Miyata H (2007). Torque teno virus (TTV): current status. *Reviews in Medical Virology*.

[39] Burián Z, Szabó H, Székely G (2011). Detection and follow-up of torque teno midi virus (“small anelloviruses”) in nasopharyngeal aspirates and three other human body fluids in children. *Archives of Virology*.

